# Hygiene of the Mouth

**Published:** 1891-07

**Authors:** J. F. Dougherty

**Affiliations:** Canton, O.


### Hygiene of the Mouth.

BY J. F. DOUGHERTY, D.D.S., CANTON, O.

Read before the Northern Ohio Dental Association at Oberlin, May 13th, 1891.

There is probably nothing in the whole range of causes, which exerts a greater influence on dentistry, than the neglect on the part of the mass of mankind, of the proper care of the teeth and mouth.

It is a surprising and shameful fact that among all grades of society there exists a woful ignorance and culpable indifference as regards the care of the teeth.

In quite a large percentage of the sufferers from deranged stomachs, neuralgia, nervous irritability, or kindred affections, the whole trouble is traceable to a neglected condition of the teeth; those organs perhaps being almost entirely hidden, and in many instances several teeth bridged over and cemented together by a thick accumulation of tartar, with a plentiful admixture of mucous, and animal and vegetable aliments, all in a state of decomposition and sending off an odor, which together with that peculiar fetor emitted by the highly congested and bleeding gums, which necessarily accompanies this condition, is sufficient to produce a breath that would do credit to a vulture that has been attending strictly to business.

Doubtless many of us are acquainted with individuals who are enthusiasts on the subject of a pure breathing medium, and who pay a great deal of attention to ventilation and sanitation generally, who at the same time are carrying about with them mouths in just such a condition as I have described, and with every inhalation take into their lungs an atmosphere as polluted as might be found in the most unsanitary habitation.

And with every ingestion of food carry into their stomachs putrefying substances dislodged from this cesspool of corruption there to continue their work of destruction.

That organs having close relationship and important physiological sympathies with the nutritive and nervous system, should, when in badly diseased condition, cause trouble in other parts, would be but a natural consequence.

It is an established medical observation that diseases of the mouth and associate parts will often cause severe morbid action in other parts of the body.

This is often the case where teeth are badly decayed and extensive accumulations of tartar have caused marked absorption of the alveolus, loosening of the teeth and general breaking up of the dental arch.

In such cases we need not be surprised to find the whole tract of the fifth pair of nerves the seat of functional derangement. And the baneful influence which such a state of things is likely to exert on the general constitution, can be appreciated only after careful consideration.

Medical men of to-day are beginning to appreciate as they never did before the very intimate relations existing between the teeth and the entire physical organization.

The ill effects following the irritation excited by neglected teeth upon the nervous system more especially, and upon the health in general is now so thoroughly recognized by every intelligent physician, that in a great many instances when called to examine a patient the condition of the teeth is enquired into as a possible cause of disease.

If, then, a neglected condition of the teeth may be the cause of so much distress and ill health, aside from the immediate and direct discomfort, how important it is that this subject be given more attention by the dental profession than it seems to have heretofore received.

The members of our profession are supposed to be quite well informed on this and kindred subjects, and to be able when called upon to instruct their patients as to the best means of maintaining perfect cleanliness of the mouth.

But alas! how many there are who impart this instruction only when it is drawn out of them by the enquiries of some earnest seeker after the truest and best means of saving their teeth; and then it is given perhaps in a careless and apathetic manner as if it occupied a very subordinate place and was scarcely worthy of serious consideration; satisfying themselves by stopping the ravages of decay by means of fillings, or substituting artificial teeth of one kind or another for the lost organs, never seeming to realize the truthfulness of the old adage, that "an ounce of prevention is worth a pound of cure" to the patient. To the dentist the cure is worth a great deal more than the prevention, hence too many of us dispense cure only, to the total exclusion of prevention.

This is a subject that receives too little attention and is treated too lightly by a great many members of our profession. This is not because its importance is not realized by every intelligent dentist, but because the initiatory step in bringing about a sanitary condition in some mouths is a very disagreeable operation and sometimes the conditions are such as to make it positively disgusting.

But however disagreeable it may be it is our duty to wade in. And when the task is finished it becomes our duty also to charge a fee commensurate to the disagreeableness of the operation.

This will have the good effect of impressing upon the minds of our patients the great importance of the operation, and stimulate them in their efforts to prevent the necessity of a frequent repetition of it.

A good fee would also have the tendency to spur us on to greater efforts in this good work. The lack of proper remuneration is too often the cause of indifference to this branch of our calling.

To bring about a sanitary condition of the mouth any diseased roots which can not be restored to a healthy condition and made suitable for carrying a crown should be extracted.

All deposits of tartar and other accumulations removed, all cavities filled, and the gums restored to a healthy condition by proper treatment. Even then the mouth can not be said to be in a sanitary condition unless the stomach be in a healthy state and performing its functions properly. For, with acid eructations from this organ, there is present in the mouth that enemy to sound teeth.

In dealing then with our patients on this subject it should be firmly impressed upon their minds that the integrity of the teeth depends very materially upon the healthy functions of the stomach; for the habitually faulty performance of the digestive process causes the dissolution of the teeth through acid reactions, as well as inducing a deterioration in their structure, from not receiving sufficient nutriment from a circulating medium rendered too poor in its nutrient elements, through dyspeptic tendencies.

A short talk to our patients on dental hygiene in general is scarcely ever ill-timed and is a subject that needs no formal introduction. We might mention the influence that diet exerts on the textures and point out the fact that certain kinds of food produce certain effects on the structures, hence the importance of a judicious choice in the articles of diet.

They should be told of the pernicious effect of certain drugs on the teeth and advised as to the proper precautions to be taken in the use of them. This is a matter to which physicians give too little attention.

The injurious effects of certain articles dispensed by street fakirs and other frauds of like character, for cleaning and whitening the teeth should be warned against. They should be taught that the decomposition of particles of food lodged between and about the teeth, forms an acid which very readily acts upon their earthy constituents and causes their early breaking down, and that in perfect cleanliness lies one of the principal means of preventing their destruction.

For this purpose nothing has ever been devised that can take the place of the tooth brush.

In selecting a brush one should be chosen of medium stiffness, not too thickly set with bristles, which are of an unequal length. With a brush of this kind, the bristles will more readily find their way between the teeth and into all the fissures just where they are most needed. The brush should be used principally in the direction of the long axis of the teeth, forcing the bristles well between the teeth and drawn toward the grinding surface. This should be done on the inside as well as on the outside of the arch. The molars and especially the third should be given particular attention.

The failure to properly clean these teeth has undoubtedly much to do with the popular notion that the wisdom teeth are not worth the efforts necessary to save them.

After meals the toothpick should be used to dislodge the coarser particles of food that have become wedged between the teeth in the masticating process. As to the kind of toothpick to be used, I would recommend either the quill or wooden article. But under no circumstances metal.

Care should be exercised in the use of the toothpick to not unduly irritate or wound the gums. Considerable harm has sometimes been done in this way.

A danger, too, in the use of the wooden toothpick is the breaking off of the end between the roots of two teeth in close apposition. Its presence there for some time may produce pericementitis and the eventual loss of the tooth. Such a case as this came under my observation not long since. The loss of a sound molar was the result.

After the toothpick should come the use of the brush as described above with tepid water. This to be followed by the use of floss silk, drawing it carefully between the teeth and over the entire proximal surface of each tooth; carrying it slightly under the free margin of the gums, all the while keeping up a sawing motion, thereby to dislodge and carry from between the teeth any small particles of food that may have escaped the toothpick aud brush. Finally, rinse the mouth well with tepid water. Either the pure article or some aromatic water. The latter is much used by the French and English, and is a habit that might with profit be imitated by their American cousins.

This cleansing process just described should be followed after eating, if that be a dozen times a day.

On arising in the morning brushing will suffice, but before retiring this might be supplemented by the rubbing of prepared chalk.

Would recommend the moderate use of dentifrices. Think harm is sometimes done by the immoderate use of preparations containing too much grit, together with unduly hard and improper brushing.

I have not hoped in the writing of this paper to advance any new thoughts, but simply to open a subject which through discussion may awaken in us a deeper interest in it and we thereby be brought to a fuller realization of our duties to our patients.

